# Ibalizumab Targeting CD4 Receptors, An Emerging Molecule in HIV Therapy

**DOI:** 10.3389/fmicb.2017.02323

**Published:** 2017-11-27

**Authors:** Simona A. Iacob, Diana G. Iacob

**Affiliations:** ^1^Carol Davila University of Medicine and Pharmacy, Bucharest, Romania; ^2^National Institute for Infectious Diseases “Prof.dr. Matei Bals”, Bucharest, Romania

**Keywords:** ibalizumab, antiretrovirals, entry inhibitors, monoclonal antibody, HIV infection, therapy

## Abstract

The HIV infection is responsible for the most devastating global pandemic of the last century. More than 39 million people have died of HIV/AIDS since 1981. The development of the antiretroviral (ARV) treatment begins with the discovery of zidovudine a nucleoside reverse transcriptase inhibitor. This breakthrough was followed by other ARV drug classes and representatives. Presently, HIV treatment employs 27 ARV representatives belonging to five different classes. Despite the proven benefits of ARV treatment and its long-term control of the HIV infection, there is an increasing concern about the numerous adverse effects and resistance to current ARV drugs. Therefore, the new HIV treatment strategies focus on the development of new ARV agents with a high genetic barrier to resistance and low toxicity. Monoclonal antibodies (MAbs) belong to a new drug class with encouraging results in the treatment of cancer, autoimmune disorders and most recently against HIV infection. The advantages of using MAbs for HIV treatment are related to their antiviral effect, lack of toxicity, good resistance profile, additional synergy with other ARV drug classes and ability to restore CD4 T-cell responses. The current article is a short summary of ibalizumab, an anti-CD4 monoclonal antibody that interferes with HIV viral entry. Current studies on ibalizumab have underlined its antiviral potential, minimal adverse effects, and lack of crossed resistance with other ARV agents thus supporting its further therapeutic use in multidrug resistant HIV-infected patients.

## Introduction

Monoclonal antibodies (MAbs) are antibodies derived from a single clone of cells able to specifically recognize and target a single antigenic epitope. Due to their high selectivity and specificity, MAbs have been successfully used in combination with conventional chemotherapeutic agents in the treatment of various autoimmune and inflammatory diseases as well as in various forms of cancer (Reichert, 2008). The encouraging results from these areas has also opened the possibility of using MAbs for the study, diagnosis, and treatment of infectious diseases (Saylor et al., 2009; Flego et al., 2013). Despite their promising perspectives in antiviral therapy and experimental approaches in various infections, only two MAbs are currently approved for use in selected cases of infectious diseases, namely palivizumab (Synagis, MedImmune, 1998) for immunocompromised high-risk infants and adults with respiratory syncitial virus infection (Hey, 2015) and bezlotoxumab (Zinplava, Merck Sharp Dohme, 2016) a human monoclonal antibody directed against Clostridium difficile toxin B for high-risk patients with recurrent disease.

The prudent approach to using MAbs in various infections is explained by the fluctuating expression of antigens on the surface of infected cells, unknown pharmacokinetics, and tissue penetration as well as the high price of MAbs compared to conventional antimicrobials (Chames et al., 2009). Nevertheless, there are a number of advantages in favor of this new biologic treatment, suggesting a beneficial use of MAbs for HIV therapy (Nakowitsch et al., 2005; Ji et al., 2007; Pugach et al., 2008; Olson and Jacobson, 2009; Saylor et al., 2009; Mould and Green, 2010; Flego et al., 2013). Thus, MAbs act on HIV-1 isolates, assist in restoring cellular immune response, exhibit a synergic effect with other antiretrovirals (ARVs) and lack significant adverse effects. Due to their low oral bioavailability, an intravenous or subcutaneous administration has been proposed instead (Ochs et al., 2006; Berger, 2011). Furthermore, the long half-lives of MAbs permit a weekly or monthly subcutaneous administration, offering a promising alternative to improve the adherence and the quality of life in HIV patients. Still, the variable pharmacokinetics of MAbs remain an important drawback for establishing the efficient doses.

Most studies on MAbs in HIV therapy or prophylaxis have been published beginning with 1993 (Posner et al., 1993; Günthard et al., 1994; Wolfe et al., 1996; Cavacini et al., 1998; Zwick et al., 2001; Armbruster et al., 2002, 2004; Khanlou et al., 2004; Kuritzkes et al., 2004; Wilkinson et al., 2005; Eda et al., 2006; Joos et al., 2006; Lalezari et al., 2006, 2008; Murga et al., 2006; Jacobson et al., 2008, 2010; Pantophlet et al., 2008; Olson and Jacobson, 2009; Song et al., 2010). Some clinical studies have addressed the safety and efficacy of different MAbs in HIV therapy, as follows: CCR5mAb004 (Human Genome Sciences, 2005—Lalezari et al., 2006; Olson and Jacobson, 2009), HGS004 (Human Genome Sciences, 2005—Lalezari et al., 2008), Pro 140 (CytoDyn, Inc., 2008–2014—Jacobson et al., 2008), F105 (NIAID, 1999—Cavacini et al., 1998), P2G12 (University of Surrey, 2011), KD 247 (The Chemo-Sero-Therapeutic Research Institute, 2009—Eda et al., 2006), as well as TNX-355 (ibalizumab). Ibalizumab is the MAb to have passed most trials as an ARV drug and is currently studied in a phase three clinical trial. It was licensed by Biogen in 1997, developed by Tanox beginning with 2004 and subsequently marketed by Taiwan TaiMed Biologics Inc. The article reviews the current literature and the most important characteristics of ibalizumab.

## General data

Ibalizumab is a human IgG4 anti-CD4 MAb derived from a murine MAb (mu5A8). The synonyms for Ibalizumab are TMB-355 (previously known as TNX-355) and hu5A8.

The development of ibalizumab was based on several studies conducted by Reimann et al. starting with 1993 (Reimann et al., 1993). Reimann observed the ability of anti-CD4 MAb mu5A8 to persist at high concentrations after repeated administrations in rhesus monkeys also covering all CD4 T cell receptors without leading to immunosuppression. Experiments with mu5A8 were renewed with humanized forms of anti-CD4 mu5A8 (hu5A8) administered to various species of macaque monkeys infected with simian immunodeficiency virus (SIV). The results suggested SIV viral suppression and a protective effect on the CD4 T cells. Additionally, hu5A8 exhibited a significantly longer plasma half-life than mu5A8 and sustained plasma levels up to 6 weeks (Reimann et al., 1997, 2002). The results strengthened the interest for these antibodies in retroviral infections. Tanox Inc. (under license from Biogen Idec) and Taimed Biologics developed TNX-355 (further renamed ibalizumab), a molecularly engineered humanized anti-CD4 MAb. In 2012 ibalizumab received FDA approval for its evaluation in phase three trials. Current studies on the subject are few (see Table 1), but results are expected to be published soon. For the moment, available data on pharmacokinetics, dosage and administration schedule are scarce.

**Table 1:** Clinical trials on ibalizumab.

Study title^*^	**Sponsor**	**Phase Trial status**	**Identifier number**
Dose-Response study of Ibalizumab (Monoclonal antibody) plus optimized background regimen in patients with HIV-1	TaiMed biologics Inc.	Phase 2 (2008–2011) completed	NCT00784147/TMB-202/ Amendment 2
Investigator-sponsored protocol–Continued use of Ibalizumab	Kaiser permanente	Phase 2 (2009–2015) Active, not recruiting	NCT01056393/5460
Compassionate use of Ibalizumab for the treatment of HIV infection	University of Colorado, Denver	Phase 3 (2012–2014) No longer available	NCT02028819
TNX-355 with Optimized Background Therapy (OBT) in treatment-experienced subjects with HIV-1	Tanox	Phase 2 (start date 2004) Unknown status	NCT00089700/TNX-355.03
Safety study of Ibalizumab subcutaneous injection in healthy volunteers prevention	TaiMed biologics Inc.	Phase 1 (2011–2012) completed	NCT01292174/TMB-108
Ibalizumab plus optimized background regimen in patient with multi-drug resistant HIV	TaiMed biologics Inc.	Phase 3 (2015–2016) completed	NCT02475629/TMB-301
Ibalizumab plus optimized background regimen in treatment-experienced patients with multi-drug resistant HIV-1	TaiMed biologics Inc.	Phase 3 (2016–2017) recruiting	NCT02707861

* *Source: http://clinicaltrials.gov, accessed May 15, 2017*.

## Mechanism of action

Ibalizumab is a genetically engineered IgG4 MAb which binds to domain 2 of CD4 T cell receptors. Ibalizumab specifically leads to conformational changes of the CD4 T cell receptor–gp120 complex thus preventing HIV fusion and entry. Therefore, ibalizumab is classified as an entry inhibitor. However, its action mechanism differs from previously known HIV entry inhibitors such as enfuvirtide (a HIV fusion inhibitor) and maraviroc (a CCR5 coreceptor antagonist).

HIV entry into the host cell's membrane is a complex multi-step process including HIV CD4 T cell receptor binding and post-CD4 binding events as follows (Kuritzkes, 2009):

1. HIV binding to CD4 T cell receptors (the attachment of HIV to the primary CD4 receptors of T helper cells). The process takes place through the interaction of the CD4 receptors with glycoprotein 120 (gp120), a trimeric molecule. This event sets in motion conformational changes of gp120 and of CD4 receptors allowing the binding of co-receptors (Moore et al., 1992).
2. HIV co-receptors binding. This process requires the interaction of gp120 and CCR5/ CXCR4 coreceptors.
3. HIV fusion with the host cell membrane mediated by glycoprotein 41.
4. HIV entry into the cell.

Each step has been investigated in controlled clinical trials as a therapeutic target for new entry ARVs. Ibalizumab inhibits post-CD4 binding events required for HIV entry (Burkly et al., 1992; Moore et al., 1992; Reimann et al., 2002; Kuritzkes, 2009; Bruno and Jacobson, 2010). It selectively binds to an epitope on the domain 2 of the CD4 receptor inducing conformational changes that ultimately prevent the interaction of gp120 and HIV co-receptors that also explain the broad spectrum of ibalizumab against CCR5 and CXCR4 isolates (Burkly et al., 1992; Kuritzkes, 2009). However, the exact site and time point when ibalizumab prevents HIV entry is not yet defined and appears to be complex. Thus, studies on viral resistance toward ibalizumab (Toma et al., 2011) suggest the presence of other action mechanisms such as conformational changes of gp120 or CD4-gp120 complex. Other data derived from the crystal structure changes of CD4 receptors following exposure to ibalizumab indicates post-co-receptor binding activity through a currently unknown mechanism (Freeman et al., 2010).

Importantly the cellular epitope targeted by ibalizumab on CD4 receptors is distant from the binding site of the major histocompatibility complex (MHC) class II molecules. This configuration prevents an MHC II mediated immune response following the interaction between ibalizumab and the CD4 receptor. As a consequence ibalizumab inhibits the post-binding entry of HIV-1 without causing immunosuppression (Moore et al., 1992; Reimann et al., 1993).

Another interesting functional characteristic of ibalizumab in comparison to other MAbs relies on its IgG4 structure. Since ibalizumab is a humananized IgG4 and IgG4 displays a low affinity for C1q and for a cellular cytotoxic dependent response (ADCC), ibalizumab will not drive to a Fc-mediated CD4^+^ T cell depletion (Michaelsen et al., 1991; Burton and Woof, 1992). The low ADCC activity of ibalizumb is a consequence of the low affinity of IgG4 for the FcɣRI receptors of NK cells. The ability of IgG4 to interact with FcɣRI relies on the sequence LEU 234-LEU 235-GLY 236-GLY 237-PRO 238-SER 239 which lies at the border of the hinge and CH2 regions of IgG. Genetic changes of this sequence (such as replacing Leu 234 with Phe) prompt either a weaker or no affinity of IgG4 for FcγRI according to Burton (Burton and Woof, 1992).

The chemical structure of ibalizumab is not public. However, considering that the preservation of CD4 T cell count is an essential requirement for the antiviral immune modulating response in HIV infection it is probable that the genetic engineering of this molecule was based on the mechanisms previously described by Burton. This hypothesis could be the case regarding to manufacture of clenoliximab, an immunomodulatoryIgG4 CD4 MAb with a similar mechanism. Clenoliximab also carries a Leu248 mutation to Glu and no Fc-dependent T cell depletion according to the results published by Reddy in 2000 (Reddy et al., 2000). Except for ibalizumab, the structure of other MAbs targeting infectious agents is based on IgG1, an immunoglobulin which exhibits its therapeutic role through Fc-mediated effector mechanisms such as complement-dependent cytotoxicity, ADCC or antibody dependent cell-mediated phagocytosis (Irani et al., 2015). Therefore, other MAbs licensed or currently under development in other medical specialties (such as rituximab, tocilizumab etc.) are based on the IgG1 structure and FcɣR-binding mechanisms to induce T cell apoptosis or block the generation of T cells pro-inflammatory cytokines (Thompson et al., 2012; Rosman et al., 2013).

In conclusion, ibalizumab is a particular anti-CD4 MAb which does not induce an immunosuppresive response, nor does it reduce the CD4 T cell count (Reimann et al., 1997; Song et al., 2010). Instead it prevents post-binding events while preserving or even increasing CD4 T cell counts as proven by *in vitro* and *in vivo* studies (Moore et al., 1992; Reimann et al., 1997; Song et al., 2010).

## Pharmakokinetics

Ibalizumab is administered via intravenous infusion or subcutaneous injection. Presently an intramuscular alternative is being evaluated (Lin et al., 2017). The average half-life of ibalizumab following subcutaneous administration is 3–3.5 days (Jacobson et al., 2009) allowing a weekly administration schedule (Bruno and Jacobson, 2010).

## Doses and efficacy

*In vitro* studies showed that ibalizumab neutralizes a broad spectrum of HIV-1 isolates, both CCR5 and CXCR4-tropic primary isolates. *In vivo* research in monkeys and humans proved that ibalizumab lowers HIV viral load while increasing CD4 T cell count (Reimann et al., 2002; Kuritzkes et al., 2004; Jacobson et al., 2009). Below are the results announced by the most prominent studies on ibalizumab.

In 2004 Kuritzkes et al. reported the results of a phase 1 trial “Antiretroviral activity of the anti-CD4 monoclonal antibody TNX-355 in patients infected with HIV type 1” (Kuritzkes et al., 2004). The study was conducted on 30 HIV patients with plasma HIV-1 RNA levels above 5,000 copies/ml and consisted of a single administration of various doses of ibalizumab. Therefore, monotherapy with a single intravenous dose of ibalizumab has prompted the following: (a) antiviral efficacy was dose dependent and was achieved by administering doses between 3 and 25 mg/kg; viral load reduction varied from 0.56 to 1.11 log_10_ copies/mL and persisted between 4 and 21 days after administration; and (b) the CD4 T cells also experience a dose-dependent increase between 23 and 244 cells/mm^3^ which persisted for 15–34 days. The preliminary study of Kuritzkes highlighted the antiviral dose-dependent and prolonged effect of ibalizumab following a monotherapy regimen, while also underlining the additional role of increasing CD4 T cells.

In 2005 and 2006 Norris et al. noted the results of a phase 2 trial “A Phase 2, multicenter, randomized, double-blind, placebo-controlled, three-arm study of the anti-CD4 monoclonal antibody TNX-355 with optimized background therapy OBR in treatment-experienced subjects infected with HIV-1 (NCT00089700)” (Norris et al., 2005, 2006). The study was performed on 82 experienced HIV infected patients resistant to multiple regimens. They were treated with intravenous ibalizumab in addition to OBR vs. placebo (only OBR) for 48 weeks. The patients were randomized to receive ibalizumab 10 or 15 mg/kg weekly for the first 8 weeks followed by either 10 or 15 mg/kg every 2 weeks vs. placebo (only OBR). After a 24**-**week follow-up in the OBR + ibaluzimab group mean viral load was reduced by 0.95–1.16 log10 copies/mL compared to a mean reduction of 0.2 log10 copies/mL in the OBR only group. The CD4 T cell count increased in the OBR+ ibaluzimab group with 9–51 cells/mm^3^ vs. OBR only 5.2 cells/mm^3^; the results were significantly better than placebo and persisted after 48 weeks.

The final mean HIV RNA reduction reported by Norris after 48 weeks of ibalizumab + OBR therapy was between 0.71 and 0.96 log_10_ copies/mL vs. a mean decrease of 0.14 using OBR. In addition the mean absolute CD4 T cell count response after a 48-week treatment increased with 48–51 cells/mm^3^ vs. only 1 cell/mm^3^ in the placebo arm. The study further proved the favorable antiviral and immune effect of ibalizumab in the group of HIV experienced patients with limited therapy options. It also confirmed the previous findings of Kuritzkes. Unfortunately after the release of these data, the full results of the trial have never been published.

In 2009 Jacobson et al. reported the results of a phase 1 trial “Safety, pharmacokinetics, and antiretroviral activity of multiple doses of ibalizumab (formerly TNX-355), an anti-CD4 monoclonal antibody, in HIV type 1-infected adults” (Jacobson et al., 2009). The study was conducted on 22 multi-experienced or naive HIV infected patients with positive but stable HIV RNA level (>5,000 copies/ml) and CD4 cell count of 100–500 cells/ml^3^. Importantly, ibalizumab was administered as monotherapy for 9 weeks and patients were subsequently followed-up for 16 weeks. The study analyzed three dosing alternatives as follows: (a) 10 mg/kg weekly, (b) 10 mg/kg loading dose followed by 6 mg/kg every 2 weeks, and (c) 25 mg/kg every 2 weeks. The *monitoring parameters* included HIV RNA plasma levels, CD4 T-cell counts, CD4 T-cell receptor coating and *in vitro* susceptibility to ibalizumab at the start and end of treatment. Most patients exhibited a transitory decline of HIV RNA level between 0.5 and 1.7 log_10_ copies/mL with peak antiviral activity within the first week. The effect of ibalizumab did not persist and viral loads returned to baseline values by the end of treatment. Moreover, reduced *in vitro* susceptibility to ibalizumab relative to baseline samples appeared in 13 of 14 patients after 9 weeks of treatment. The most remarkable increase in CD4 T-cell counts was recorded at week one and involved an increase of 112 cells/μL from the baseline following a weekly dose of 10 mg/kg. However, all study arms were followed by a progressive decrease in CD4 T cell counts with values that stabilized around the baseline at the end of the treatment period. The coating of CD4 T-cell receptors was dependent on serum ibalizumab concentration. At the end of treatment, 13 out of 14 patients displayed a reduced percentage of maximum inhibition toward ibalizumab. Still, no mutations were found in relation to ibalizumab resistance after 9 weeks of treatment and the strains remained sensitive to enfuvirtide. These results proved the dose-dependent antiviral effect of ibalizumab and its role in preserving the CD4 T cell counts. On the other hand, the study highlighted the decreasing susceptibility that occurs during treatment with ibalizumab and discouraged the administration of ibalizumab in monotherapy. Regarding the safety and pharmacokinetics ibalizumab was well-tolerated and well-accepted by patients, while the level of anti-ibalizumab antibodies was minor and did not significantly change the serum level of ibalizumab.

In 2011 Khanlou et al. added the results of a phase 2 study “Safety, efficacy, and pharmacokinetics of ibalizumab in treatment-experienced HIV-1 infected patients: a phase 2b study” (Khanlou et al., 2004). The study was performed on 113 multiresistant HIV-1 infected patients treated with ibalizumab and OBR for 24 weeks vs. a control group (only OBR) for comparison. Ibalizumab was administered intravenously in two doses of 800 mg q2wks (variant 1) or 2,000 mg q4wks (variant 2). Finally the viral load showed a significant decrease of 1.5–1.6 log_10_copies/ml; the mean increase in the CD4 T cell count after 24 weeks varied between 37 and 40 cells/μL. No side effects were reported.

Preliminary results of a major phase 3 trial namely “Ibalizumab plus optimized background regimen in treatment-experienced patients with multi-drug resistant HIV-1” (NCT02475629/TMB-301) were communicated in 2017. The study was performed on 40 patients with virologic failure under ARV treatment exhibiting mean viral loads of >1,000 copies/ml and a mean CD4 T cell count of 150 cells/μL. The primary endpoint was the viral load reduction at 14 days after ibalizumab monotherapy with a loading dose of 2,000 mg iv; the secondary endpoints focused on the proportion of patients able to sustain an undetectable viral load and a CD4 T cell count increase as well as a satisfactory safety and tolerability profile throughout 24 weeks of treatment. The study disclosed a good tolerability and high efficacy even in patients experiencing virologic failure to more than 10 ARV agents (Lewis et al., 2017).

The current literature results on ibalizumab are still limited and precludes a definitive conclusion on the administration schedule or best dosage for ibalizumab. Nevertheless, trials have pointed to an important correlation between the therapeutic effectiveness and intravenous dose suggested mostly by Khanlou and Kuritzkes (Khanlou et al., 2004; Kuritzkes et al., 2004).

Preexposure prophylaxis using ibalizumab was the focus of the study NCT01292174/TMB-108 (TaiMed Biologics Inc.). The trial involved a weekly subcutaneous administration of various doses (120, 240, and 480 mg) in HIV-negative volunteers but results have not yet been disclosed. Further studies on the subject also need to address the bioavailability of ibalizumab, the concentrations in the genital secretions and the potential prophylactic use of ibalizumab for a subcutaneous monthly administration.

To facilitate the study of ibalizumab, the US FDA approved in May 2014 the manufacture of ibalizumab drug substance in China by WuXiPharmaTech.

## Resistance

Primary resistance to ibalizumab is estimated at around 10%. Secondary resistance could ensue after the omission of a single dose according to Fessel (Godofsky et al., 2005) as well as after 9 weeks of ibalizumab monotherapy according to Jacobson (Fessel et al., 2011). Resistance to ibalizumab leads to a highly infectious viral strain without developing concomitant resistance to other HIV entry inhibitors namely enfuvirtide (a fusion inhibitor) and maraviroc (a CCR5 antagonist) (Zhang et al., 2006; Jacobson et al., 2009).

Reduced susceptibility to ibalizumab ensues if HIV-1 loses a glycan in the N-terminus of gp 120 V5 (Toma et al., 2011; De Feo and Weiss, 2012; Pace et al., 2013a). Therefore, the susceptibility to ibalizumab could be restored by placing a glycan molecule in the variable region of the antibody (Song et al., 2013). An alternative could be a new generation of humanized monoclonal recombinant antibodies (TMB-360 and TMB-365) obtained through genetic engineering, currently studied by a group of researchers at Aaron Diamond AIDS Research Center. This new generation is a glycan-modified variant of ibalizumab, with a superior pharmacological profile and improved stability (Pace et al., 2013a). Such variants with an improved pharmacokinetic/pharmacodynamic profile requiring fewer doses could be proposed in the future through the addition of long acting ARV drugs in patients with particularly low adherence to oral regimens.

## Side effects

Previous studies have proved that ibalizumab is safe; side effects after intravenous administration are rare, yet mild to moderate and dose-dependent, usually resulting in rash (14–15%), diarrhea (0–14%), headache (7–11%), nausea (4–11%), and depression (4–11%) (Khanlou et al., 2004; Norris et al., 2006). Severe (grade 3) laboratory abnormalities were seen in 9–10% of cases during a 48-week treatment. Additional life-threatening (grade 4) laboratory results were found in only 2–4% of cases, compared with 5% in patients receiving placebo. No drug-related deaths or discontinuations occurred in the above mentioned studies. The variations of common laboratory parameters were not clinically relevant. The intramuscular administration was also safe and well-tolerated without local side effects at the injection site.

## Future perspective

Ibalizumab is currently under review by the FDA following the acceptance of a Biologics License Application on June 30, 2017.

Current results indicate that ibalizumab has a broad-spectrum activity against HIV isolates, including particularly resistant strains, while also preserving CD4 T cell counts. Furthermore, its safety profile allows its use as part of a combination regimen with other ARV drugs, including enfuvirtide (Zhang et al., 2006; Jacobson et al., 2009). The most recent data published in NCT02475629 study (Irani et al., 2015) confirms the virologic efficacy and safety of ibalizumab + OBR as a salvage regimen in multidrug resistant HIV patients. It is thus hoped that ART regimens containing ibalizumab could be successfully adopted by experienced patients with limited or no other therapeutic options. The cost effectiveness of a salvage therapy with ibalizumab, a new and expensive entry inhibitor remains to be established.

Another extremely promising indication of MAbs is the antibody-drug conjugates (ADCs), a new line of therapeutic agents presently used in the treatment of cancer and lymphomas, which hopefully will be translated into HIV treatment (Alley et al., 2010). Current preclinical studies have employed ADCs molecules containing either ibalizumab or ibalizumab derived molecules attached to other ARV molecules and conjugated with histone deacetylase inhibitors, a class of compounds that reverse HIV latency (TaiMed Biologics). Such complex molecules able to activate the integrated silent proviral HIV DNA and to eliminate HIV infected cells could become a first line therapeutic regimen in HIV patients. The combination therapy with ibalizumab and anti-gp120 (bispecific antibodies) also shows promise as a prophylactic HIV preexposure regimen according to studies conducted by Pace et al. (2013b) and Song et al. (2016).

The use of ibalizumab in prophylactic regimens has also been considered. Pre-exposure prophylaxis (PrEP) is an effective strategy of lowering the risk of HIV transmission by as much as 92% in categories with the highest risk of HIV transmission, namely injectable drug users and people with sexual exposure to HIV (sero-discordant couples and homo or heterosexual persons who do not use condoms or other protection methods). Notably, PrEP is the only means of protection in these group categories in the absence of a vaccine. The only regimen currently approved for PrEP implies the use of tenofovir and emtricitabine according to several administration schedules as described in the guidelines. Despite the efficiency of the current PrEP drugs, this regimen entails a series of disadvantages such as the difficulty to attain an adequate adherence, the celullar mechanism by which these ARVs act only after HIV entry into the cell and various side effects. Ibalizumab could prove an important alternative considering the following points: the action mechanism (entry inhibitor), a favorable safety profile with few side effects and the long half-life which enables a weekly administration and enhances the adherence to prophylaxis. On the other hand the pharmacologic properties of ibalizumab need to be clarified including—the tissue distribution (in particular the genital tract and rectum), the risk of resistance and the adequate dosing schedules for various prophylactic strategies. The potential use of ibalizumab for PrEP depends on results of the current pharmacokinetic and pharmacodynamics studies, as well as on the production costs and comparative advantages and disadvantages with other ARVs awaiting approval (such as LA-rilpivirine or GSK1265744). A phase 1 study initiated in February 2011 (NCT01292174) on the safety and tolerability of ibalizumab as a weekly subcutaneous injection could offer more interesting data on this indication. In 2010 the CAVD group (The Collaboration for AIDS Vaccine Discovery) in collaboration with TaiMed Biologics also announced a Phase 1 clinical trial on the role of ibalizumab in HIV prevention. However, data from these studies has yet to be published.

## Conclusion

Ibalizumab is an experimental monoclonal antibody with significant anti HIV-1 activity and minor adverse effects currently undergoing phase 2 and 3 trials. The exact action mechanism of ibalizumab remains to be elucidated. Preliminary data have consistently shown that ibalizumab induces conformational changes of the CD4 receptors and gp120 that prevent post-CD4 binding events without eliciting an immunosuppressive MHC II response. Ibalizumab can be administered both intravenously and subcutaneously with a satisfactory effect on decreasing HIV RNA serum levels also preserving CD4 T cell counts. As a result of its ability to block the entry of HIV-1 multi-resistant isolates, ibalizumab has been studied in combination with other ARV drugs with favorable results in experienced patients. On the other hand various clinical trials are currently examining the subcutaneous administration of ibalizumab as a single agent for HIV prevention. Still, further data is needed to establish the most efficient doses, drug interactions and potential ARV combinations.

While the definitive indications for ibalizumab yet to be established, ibalizumab will probably become a niche product as part of a salvage regimen for the most vulnerable category of HIV patients, those with extensive drug resistance.

## Author contributions

SI and DI had an equal contribution to the manuscript (acquisition, interpretation, and revising of data) and agreed upon the final version of the manuscript. Both authors agree to be accountable for all aspects of accuracy or integrity of this work.

### Conflict of interest statement

The authors declare that the research was conducted in the absence of any commercial or financial relationships that could be construed as a potential conflict of interest.
